# Analysis of epigenetic changes in survivors of preterm birth reveals the effect of gestational age and evidence for a long term legacy

**DOI:** 10.1186/gm500

**Published:** 2013-10-18

**Authors:** Mark N Cruickshank, Alicia Oshlack, Christiane Theda, Peter G Davis, David Martino, Penelope Sheehan, Yun Dai, Richard Saffery, Lex W Doyle, Jeffrey M Craig

**Affiliations:** 1 Early Life Epigenetics Group, Murdoch Childrens Research Institute (MCRI), Royal Children’s Hospital, Flemington Road, Parkville, Victoria 3052, Australia; 2 Department of Paediatrics, University of Melbourne, Royal Children’s Hospital, Flemington Road, Parkville, Victoria 3052, Australia; 3 Present address: Telethon Institute for Child Health Research, University of Western Australia, 100 Roberts Road, Subiaco, WA 6008, Australia; 4 Bioinformatics Group, MCRI, Royal Children’s Hospital, Flemington Road, Parkville, Victoria 3052, Australia; 5 Neonatal Services, Royal Women’s Hospital, Parkville, Victoria 3052, Australia; 6 Department of Obstetrics and Gynaecology, University of Melbourne, Royal Women’s Hospital, Parkville, Victoria 3052, Australia; 7 Cancer and Developmental Epigenetics Group, MCRI, Royal Children’s Hospital, Flemington Road, Parkville, Victoria 3052, Australia

## Abstract

**Background:**

Preterm birth confers a high risk of adverse long term health outcomes for survivors, yet the underlying molecular mechanisms are unclear. We hypothesized that effects of preterm birth can be mediated through measurable epigenomic changes throughout development. We therefore used a longitudinal birth cohort to measure the epigenetic mark of DNA methylation at birth and 18 years comparing survivors of extremely preterm birth with infants born at term.

**Methods:**

Using 12 extreme preterm birth cases and 12 matched, term controls, we extracted DNA from archived neonatal blood spots and blood collected in a similar way at 18 years of age. DNA methylation was measured at 347,789 autosomal locations throughout the genome using Infinium HM450 arrays. Representative methylation differences were confirmed by Sequenom MassArray EpiTYPER.

**Results:**

At birth we found 1,555 sites with significant differences in methylation between term and preterm babies. At 18 years of age, these differences had largely resolved, suggesting that DNA methylation differences at birth are mainly driven by factors relating to gestational age, such as cell composition and/or maturity. Using matched longitudinal samples, we found evidence for an epigenetic legacy associated with preterm birth, identifying persistent methylation differences at ten genomic loci. Longitudinal comparisons of DNA methylation at birth and 18 years uncovered a significant overlap between sites that were differentially-methylated at birth and those that changed with age. However, we note that overlapping sites may either differ in the same (300/1,555) or opposite (431/1,555) direction during gestation and aging respectively.

**Conclusions:**

We present evidence for widespread methylation differences between extreme preterm and term infants at birth that are largely resolved by 18 years of age. These results are consistent with methylation changes associated with blood cell development, cellular composition, immune induction and age at these time points. Finally, we identified ten probes significantly associated with preterm individuals and with greater than 5% methylation discordance at birth and 18 years that may reflect a long term epigenetic legacy of preterm birth.

## Background

Preterm birth, defined as birth earlier than 37 weeks of gestation, is a major cause of neonatal death. Moreover, preterm birth imposes substantial health burdens on survivors; for example, children born preterm are four to five times more likely to develop brain and cardiovascular disorders compared with infants born at term [1,2]. As a group, preterm survivors are at increased risk for chronic illnesses later in life relating to respiratory [3,4], visual [5], cardiovascular [6], hearing [7,8] and intellectual/behavioral [9,10] impairment. The molecular mechanisms that might confer increased risk on these complex traits are incompletely understood.

Preterm birth imposes stress on infants due to premature removal from the intrauterine environment. Environmental factors relevant to preterm birth, such as nutrition, temperature change, toxins, and hypoxia/hyperoxia (that is, stressors) can alter gene expression in the short and/or long term [11,12]. In mice, maternal nutritional status *in utero*[13-15], or maternal care during post-natal development [16-18] are associated with stable epigenetic alterations in the offspring (such as DNA methylation and histone post-translational modifications) accompanied by metabolic or behavioral alterations. Other studies have shown that long-term effects of gestational hypoxia in a mouse model may be independent of lasting epigenetic alterations, but dependent on gene-environment interactions [19]. These persistent, environmentally induced phenotypic alterations have been attributed to aberrant organ development following transiently disrupted cell signaling [19]. Thus, mechanisms mediating long-term phenotypic variation in response to early environment remain controversial.

Genomic regions subject to DNA methylation change have been identified during gestation [20-22], neonatal development [23] and the entire lifespan [24-28]. The aim of this exploratory study was to assess genome-wide DNA methylation profiles of extremely preterm survivors compared with term controls at both birth and at 18 years of age, using a longitudinal case-control study design.

## Methods

### Ethics approval

The study was approved by the Human Research Ethics Committees of the Royal Women’s Hospital and the Royal Children’s Hospital (Melbourne) and conformed to the Helsinki Declaration.

### Subjects

The subjects of this study were 12 preterm infants born at less than 31 weeks of gestational age and 12 term controls. All were born at the Royal Women’s Hospital, Melbourne in 1991 or 1992, and enrolled in a longitudinal study from birth. They were derived from a list of 18 pairs of subjects who consented (parents and subjects) to participate in the study and provide neonatal Guthrie cards (GCs) and 18-year dried blood spots. Subjects were matched for sex, ethnicity (all Caucasian), and singleton birth status. Gestational age (GA), sex, and delivery modes of subjects are shown in Table 1.

**Table 1:** Demographic, clinical and sample characteristics of the study cohort

	**Preterm probands**	**Term probands**
Gestational age, weeks^a^	26 (25 to 30)	39 (36 to 42)
Age at Guthrie card birth sample, days^a^	6.5 (5 to 20)	4.0 (4 to 5)
Sex, male^b^	8/12 (67%)	9/12 (75%)
Born by Cesarean section^b^	3/12 (25%)	8/12 (33%)
Mother experienced labor^b^	9/12 (75%)	10/12 (83%)

^a^Median (range).

^b^Number/total number (%).

### DNA extraction and bisulfite conversion from dried blood spots

Six to ten 3 mm punches taken from dried blood spots were placed in a 1.5 ml tube with 100 μl water with a single tungsten carbide bead (3 mm; catalog number 69997; Qiagen, Victoria, Australia). Samples were macerated with a TissueLyser II (Qiagen) (time: 5 × 30 seconds, frequency: 30 Hz). Beads were removed, and samples were further processed using the QIAamp DNA Mini Kit (Qiagen), in accordance with the manufacturer’s instructions, but with the following modifications. The macerated sample was incubated with 190 μl of extraction buffer (ATL; SDS-containing proprietary formula extraction buffer; Qiagen) for 10 minutes at 85°C for 10 minutes. Supernatant was collected and the ATL extraction of the homogenate was repeated twice. The resulting extract was pooled and further processed by proteinase K digestion (60 μl; 10 mg/ml at 56°C for 1 hour), then incubated with 600 μl buffer AL for 10 minutes at 70°C, followed by addition of 600 μl 100% ethanol. After mixing by inversion, samples were loaded onto a single QIAamp column by repeat centrifugation. Following washing, DNA was collected by incubating twice with 100 μl buffer AE (10 mM Tris-HCl, 0.5 mM EDTA, pH 9.0) for 10 minutes, and once with 50 μl buffer AE for 10 minutes. The combined eluates were placed in a Speedvac at 45°C for 8 hours. The dry pellet was resuspended in 40 μl TE (10 mM Tris-HCl, 1 mM EDTA, pH 8.0) and quantified by spectrophotometry (Nanodrop, Wilmington, DE, USA) yielding a median of 1.7 μg DNA. Residual homogenates from a subset of samples were subjected to the same extraction process twice, yielding an additional 1.6 μg DNA. DNA samples (1 μg) were bisulfite-converted using the MethylEasy Xceed bisulphite conversion kit (Human Genetic Signatures, North Ryde, Australia), in accordance with the manufacturer’s instructions. Conversion efficiency was confirmed by bisulfite-specific PCR [29].

### Infinium methylation analysis

We used the Illumina Infinium HumanMethylation 450 (HM450) BeadChip platform, which interrogates more than 485,000 CpG dinucleotides, with probes targeted to CpG islands (CGIs), and their adjacent shores (2 kb regions flanking CGIs) and shelves (2 kb regions flanking shores); non-coding RNA; gene promoters, enhancers, and intergenic regions; and regions associated with epigenetic reprogramming of fibroblasts to inducible pluripotent stem cells (reprogramming-specific differentially methylated region; rDMR) [30,31]. Bisulfite treated DNA was hybridized to HM450 BeadArrays, with both birth and 18-year samples from three preterm and three term probands (total of twelve samples) selected per array in a scrambled order by ServiceXS (Amsterdam, The Netherlands).

Raw intensity data (IDAT) files were imported into the R environment (version 2.14.1) [32] using the *minfi* package [33]. Data quality was assessed with plots derived from various control probes on the array. Probes from the X and Y chromosomes (n = 11,648) were removed. Probes were excluded if they failed in one or more samples based on a detection *P*-value of greater than 0.01 (n = 96,632). This method will remove any probes that might correspond to degraded regions of the genome from long-term storage of the samples at room temperature. One term birth sample with mean detection *P* > 0.05 was excluded from analysis. The data were pre-processed using the Illumina method (bg.correct = ‘FALSE’, normalize = ‘controls’) and subset-quantile within-array normalization (SWAN) was performed [34]. Probes targeting CpG dinucleotides containing a known single nucleotide polymorphism (SNP) and HM450 control probes were excluded from analyses (n = 23,365). The resulting dataset comprised 347,789 autosomal probes from 11 term and 12 preterm birth samples, and 12 term and preterm 18-year samples. The log_2_ ratio of methylated probe intensity to unmethylated probe intensity was calculated in *minfi*, denoted as M-values used for statistical analyses, and converted to β values ranging from 0 to 1 (0 to 100% methylation) [35,36]. The HM450 data are available from Gene Expression Omnibus (GEO) with an accession number of GSE51180.

### Statistical analysis

The data underwent unsupervised hierarchical clustering analysis and multi-dimensional scaling (MDS) using lumi [35]. Heatmaps and dendrograms were drawn with *gplots*[37]. Differential methylation analysis was performed on M-values using the *limma* package [38] setting the false discovery rate (FDR) cut-off point at less than 0.05 using the Benjamini-Hochberg procedure [39]. Correlation of methylation values at birth and 18 years across individuals was assessed using the *duplicateCorrelation* function [40]. For differential analysis, a linear model was fitted with age, case-control status (preterm or term), and predictive factors correcting for sex and array effects. Differentially methylated genes were determined if any probe associated with the gene was called ‘differentially methylated’. Gene ontology enrichment was performed using the DAVID bioinformatics tool under the default settings [41,42] and pathway analysis using Ingenuity Pathways Analysis (IPA) software (Ingenuity Systems, Redwood City, CA, USA). Differentially methylated probes (DMPs) were classified as gene-related, CGI-related [43], DMRs [44], or regulatory regions (promoters, enhancers, and DNAse hypersensitivity sites). Enrichment and gene set tests were populated with probe IDs using annotations provided in the Illumina HM450 manifest (version 1.2). Gene lists were consolidated by replacing multiple isoforms (for example, *Protocadherin* genes) with a single RefSeq entry, or including multiple RefSeq entries associated with a single probe where bidirectional gene loci (for example, *ABI3* and *GNGT2*) or host gene/non-coding RNA genes (for example, *ITPR1* and *EGOT*) were identified. The limma function *decideTests* was used to identify directional correlations (method = ‘separate’; adjustment method = ‘BH’; and *P* = 0.05) and visualized with *heatDiagram*. Genomic location enrichment was determined by calculating the ratios of observed/expected (O/E) probes in each category, and classified as over-represented (O/E ratio >1) or under-represented (O/E ratio <1), with significance assigned using hypergeometric means tests (statistics package: *phyper* function, one-sided lower tail for under-representation or one-sided upper tail for over-representation). Significance of birth/DMP and age/DMP overlap was assessed using Fisher’s exact test for count data (statistics package: *fisher.test*).

### Sequenom MassArray target validation

Target validation was performed using the Sequenom MassArray EpiTYPER (Sequenom, San Diego, USA), performed as previously described [21,29]. Amplicons were designed using the Sequenom EpiDesigner [45] and MassArray [46], and tested *in silico* using methBlast [47] software. Oligonucleotide sequences were prepared (see Additional file 1: Table S1) such that forward primer sequences contain a 10 bp tag (AGGAAGAGAG) at their 5′ ends, and reverse primer sequences contain a 31 bp tag (CAGTAATACGACTCACTATAGGGAGAAGGCT) at their 5′ ends. Amplification was performed using 1 μl bisulfite-converted DNA with the FastStart kit (Roche, Mannheim, Germany) in 15 μl reactions with thermocycling conditions as follows: 94°C for 2 minutes; 5 cycles of 94°C for 30 seconds, 60°C for 30 seconds, and 72°C for 30 seconds; 35 cycles of 94°C for 30 seconds, 62°C for 30 seconds, and 72°C for 30 seconds; and final elongation at 72°C for 6 minutes. Data processing was carried out in triplicate using the median methylation level at specific CpG sites. Raw data obtained from MassArray EpiTYPING were cleaned systematically using an R-script to remove samples that failed to generate data for more than 70% of the CpG sites tested. In addition, technical replicates showing 10% or greater absolute difference from the median value were removed, and only samples with at least two successful technical replicates were analyzed.

## Results

### An improved method of DNA extraction from Guthrie cards

We used a bead-facilitated maceration method involving repeat extractions, tested for applicability for Infinium HM450 arrays [48]. DNA from archived GCs sampled at birth and at 18 years of age yielded a median of 1.6 μg DNA after the first extraction, increasing to 3.3 μg DNA after two additional rounds of extraction using six to ten 3 mm blood spots. Greater amounts of DNA were recovered than previously reported [49-52], and were similar using blood spots stored desiccated at room temperature for 1 or 18 years (data not shown).

### Exploratory analysis of components of epigenetic variation

The characteristics of our study cohort and sample collection are summarized in Table 1. MDS identified age (birth versus 18 years) as the predominant source of variation within the dataset (see Additional file 2: Figure S1). We noted increased inter-individual variability between birth samples held in long-term storage (see Additional file 2: Figure S1; also data not shown). However, the similarity of β-value distributions of birth and 18-year samples (see Additional file 2: Figure S2) and the probe intensity of control probes (data not shown) indicated similar performance of these samples on the HM450 platform. To explore other components of variation, we tested associations of sex, delivery mode (spontaneous or iatrogenic; vaginal or lower uterine cesarean section; and labor or induced) and array. Sex and array were identified as significant factors, and were used as explanatory variables in linear models for subsequent analyses. Probes associated with sex included several autosomal loci homologous to X or Y chromosomes (data not shown), probably reflecting cross-hybridization, as previously reported [53].

### Identification of gestational age-associated differential methylation at birth

**Figure 1:**
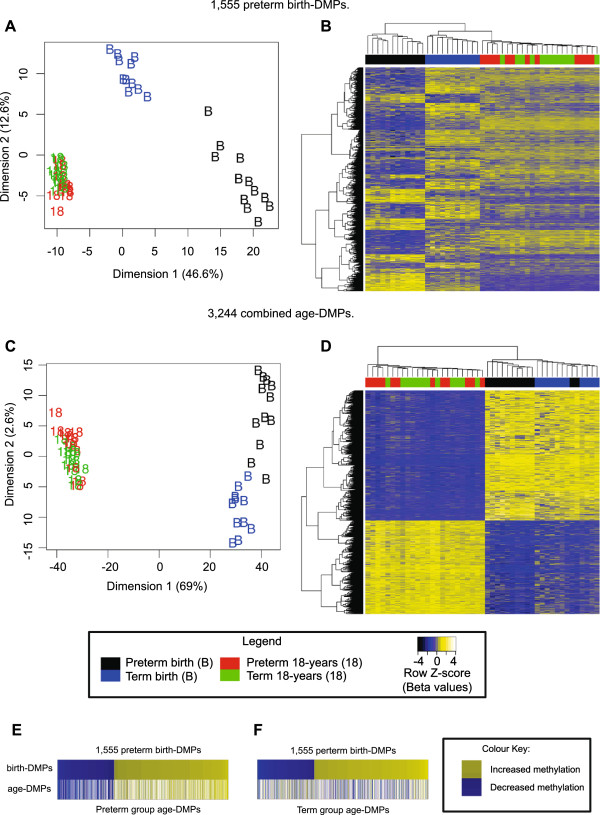
**Preterm-associated differentially methylated probes (DMPs) at birth overlap with age-associated DMPs. (A)** Multi-dimensional scaling (MDS) and **(B)** heatmap plots of 1,555 significant preterm birth DMPs (comparing preterm birth samples with term birth samples). **(C)** MDS and **(D)** heatmap plots of 3,244 combined age DMPs with β > 0.2 (comparing all birth samples with all samples obtained at 18 years). **(E,F)** Heat diagrams displaying all birth DMPs (upper rows) with probes colored by the direction of methylation change from preterm to term, either increasing (yellow shading) or decreasing (blue shading). Comparison of overlap of birth DMPs and age DMPs determined using either **(E)** preterm or **(F)** term subjects showed a high proportion of sites that differed in a similar direction when assessing methylation changes with age in preterm subjects (934 probes changed in the same direction; 34 probes changed in the opposite direction). By contrast, methylation changes with age in term subjects showed more sites that differed in the opposite direction with age (300 probes changed in the same direction; 431 probes changed in the opposite direction). The sample groups are color coded as follows: term birth, blue; preterm birth, black; term birth at 18 years, green; preterm at 18 years, red.

We tested for differential methylation between preterm and term birth samples and identified 1,555 DMPs (birth DMPs; FDR < 0.05) (Figure 1A,B; see Additional file 3: Table S2). Cross-platform validation was performed using Sequenom EipiTYPER assays targeting CpG sites near DMPs, because the methylation profiles of CpG sites in close proximity are highly correlated [54]. Specifically, we tested DNA methylation within the first intron of the *VWF* gene as a representative enhancer site birth DMP, which is known to regulate gene expression [55] (two probes, Pearson *r*^2^ = 1.000, *P* < 0.012; r^2^ = 0.954, *P* = 0.023 across all samples) (see Additional file 2: Figure S3). In agreement with two previous studies of DNA methylation associated with GA, we observed birth DMPs at gene loci encoding the transcription factor nuclear factor I/X (*NFIX,*[20]), oxytocin (*OXT*), and arginine vasopressin (*AVP*) [22].

Gene ontology classes associated with birth DMPs showed a bias towards biological processes involved in GTPase signaling (for example, *PLEKHG5*, *RASA3*, and *AGAP1*), transcription (for example, *LEF1*, *DNMT3A*, and *NCOR2*), embryonic morphogenesis for example, *WNT3A*, *NODAL*, and *SHANK3*), cell growth and proliferation (for example, *RUNX1*, *BMP1,* and *DOT1L*), and nervous system (for example, *FGF1*, *GABBR1*, and *GDNF*) and hematological (for example, *AIRE*, *IL12A*, and *PBX1*) development (see Additional file 4: Table S3; DAVID ontology). Pathway analysis showed over-representation of antigen presentation pathway genes (see Additional file 4: Table S3; IPA ‘Pathway’). Analysis of upstream regulators of genes associated with birth DMPs found a significant overlap with genes regulated by the transcription factor CREB1 and the Ca^2+^/calmodulin-dependent protein kinase complex, CaMKII (*P* < 10^-4^) (see Additional file 4: Table S3; IPA ‘Upstream’).

### Age-related changes in DNA methylation overlap with birth DMPs

We next sought to identify probes that differ between whole blood from infants and 18-year-olds. We compared all birth samples with all 18-year samples, independent of preterm status (birth, n = 23; 18 years, n = 24). Interestingly, we found no overall evidence for a correlation within individuals between the two time points (consensus correlation; *r* = −0.0343). We identified 116,603 age-associated DMPs (‘age DMPs’, adjusted *P* < 0.05) including 3,244 probes with mean DNA methylation (β) change greater than 0.2 (Figure 1C,D; see Additional file 5: Table S4).

Because age DMPs clustered the birth samples according to preterm/term status (Figure 1C), we tested the hypothesis that epigenetic change is continuous from mid-gestation to 18 years of age; that is, that birth DMPs and age DMPs would share common probes. To account for methylation changes occurring during gestation, we performed analyses using birth and 18-year samples from the term group (birth, n = 11; 18 years, n = 12) and preterm group (birth, n = 12; 18 years, n = 12) separately. Comparing birth DMPs with age DMPs as defined from preterm subjects (n = 56,515 probes), we found a continuum of change comprising 934 of 1,555 (60%; *P* < 2.2 × 10^-16^, odds ratio (OR) = 7.76) sites of methylation difference that were also differentially methylated in the same direction by 18 years of age, and 34 of 1,555 (2%; *P* < 2.2 × 10^-16^, OR = 0.11) sites that had changed in the opposite direction by 18 years age (Figure 1E). By contrast, comparing birth DMPs with age DMPs defined from term subjects (n = 63,127), we identified 300 of 1,555 (19%; *P* < 2.2 × 10^-16^, OR = 1.08) probes that were directionally correlated, and 431 of 1,555 (28%; *P* < 2.2 × 10^-16^, OR = 1.73) probes that were differentially methylated in the opposite direction at 18 years (Figure 1F).

**Figure 2:**
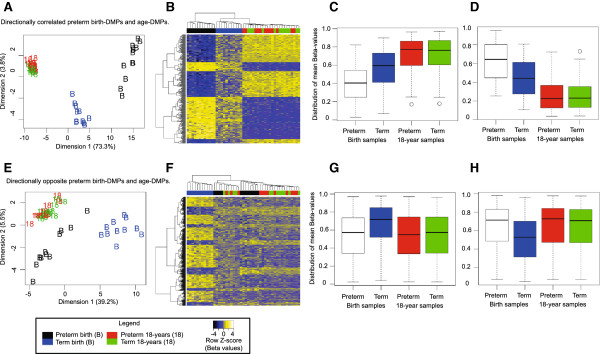
**Comparison of correlated and directionally opposed birth and age differentially methylated probes (DMPs). (A)** Multi-dimensional scaling (MDS) and **(B)** heatmap plots of 300 directionally correlated birth DMPs. Mean group β-values of correlated DMPs that **(C)** increased (159 probes) or **(D)** decreased (141 probes) with age. **(E)** MDS and **(F)** heatmap plots of 431 directionally opposed age and birth DMPs. Mean group β-values of directionally opposed DMPs that were **(G)** higher (314 probes) or **(H)** lower (117 probes) in term birth samples. The sample groups are color coded as follows: term birth, blue; preterm birth, black; term birth at 18 years, green; preterm at 18 years, red. Birth DMPs and age DMPs (defined using term group only) were analyzed by sub-setting probes that had changed in the same (correlated) or differing directions (opposing).

We further investigated the overlap of birth DMPs and age DMPs by cluster analysis, and found that preterm birth samples appeared as a sub-group distinct from the term birth and 18-year samples (Figure 2A,B) using directionally correlated probes. By contrast, using the directionally opposed probes, we found that term birth samples appeared as a separate group in cluster analysis (Figure 2E,F) and in plots of the distribution of mean β-values (Figure 2G,H).

We found that birth DMPs and age DMPs showed similar ontology and pathway enrichments (see Additional file 6: Table S5). These included GTPase signaling, transcription and embryonic morphogenesis, nervous system and hematological system development, and the antigen presentation pathway. Transcription factors were identified as predominant upstream regulators of genes associated with age DMPs, with a significant overlap of genes regulated by NLRC5, NKX2-3, and FOXC1 (*P* < 10^-4^) (see Additional file 6: Table S5). Upstream pathway analysis of genes that showed a continuum of methylation change from preterm birth to 18 years (directionally correlated probes) showed enrichment for genes regulated by the transcription factors NLRC5, CIITA, and PML (*P* < 10^-4^) (see Additional file 7: Table S6), whereas genes that showed methylation change in the opposing direction were over-represented by genes regulated by the transcription factors MTA1, JUN, and TP53 (*P* < 10^-4^) (see Additional file 8: Table S7).

**Figure 3:**
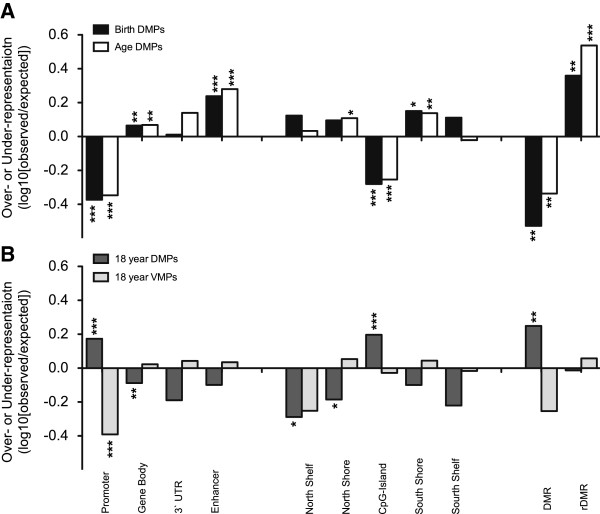
**Genomic and sequence context of differentially methylated probes (DMPs).** Enrichment or depletion of DMPs displayed as the log_10_ of the observed/expected frequencies for each category with significance from hypergeometric test results displayed as *P < 10^-5^, **P < 10^-10^, and ***P < 10^-20^. **(A)** Genomic enrichment distribution of 1,555 birth DMPs (black) and 3,244 age DMPs (white) showed similar profiles. **(B)** Top 1,500 probes ranked by odds of differential methylation at 18 years (18-year DMPs; dark grey) and the top 1,500 most variable methylated probes (18-year VMPs; light grey) showed distinct enrichment profiles, with the 18-year DMPs showing the opposite enrichment to the birth DMPs and age DMPs.

To determine whether age DMPs and birth DMPs were enriched at similar genomic regions (for example, gene-associated regions, regions with proximity to CGI) or regulatory functions (for example, promoters, enhancers), we performed an enrichment analysis of birth DMPs with contextual data supplied with the HM450 arrays. Birth DMPs and age DMPs also showed similar genomic context biases (Figure 3A). We found that promoters (birth DMPs: 0.4-fold, *P* = 5.8 × 10^41^; age DMPs: 0.4-fold, *P* = 1.7 × 10^-75^) and CGIs (birth DMPs: 0.5-fold, *P* = 5.0 × 10^-55^; age DMPs: 0.5-fold, *P* = 1.2 × 10^-97^) were both under-represented. By contrast, enhancers were over-represented (birth DMPs: 1.7-fold, *P* = 5.6 × 10^-37^; age DMPs: 1.9-fold, *P* = 2.6 × 10^-111^), as to a lesser extent were gene bodies (birth DMPs: 1.2-fold, *P* = 1.2 × 10^-6^; age DMPs: 1.2-fold, *P* = 3.2 × 10^-13^). We also tested enrichment at rDMRs, and observed significant enrichment (birth DMPs: 2.3-fold, *P* = 4.0 × 10^-12^; age DMPs: 3.4-fold, *P* = 7.0 × 10^-66^).

To test if such concordance was related to probe variability or potential statistical/array artifacts, we compared genomic context enrichment with two probe sets identified using 18-year DNA methylation profiles. We used the top 1,500 most variable methylated probes (VMPs) across all 18-year samples and the top 1,500 probes ranked by odds of differential methylation, comparing 18-year DMPs for both preterm and term groups. We found that these sets of probes showed unique genomic context distributions compared with age DMPs and birth DMPs, suggesting a biological rather than technical basis for genomic context profiles (Figure 3A,B). Notably, top-ranked 18-year DMPs showed the opposite genomic context profiles compared with birth DMPs and age DMPs.

### Evidence for a long-term legacy of prematurity

**Figure 4:**
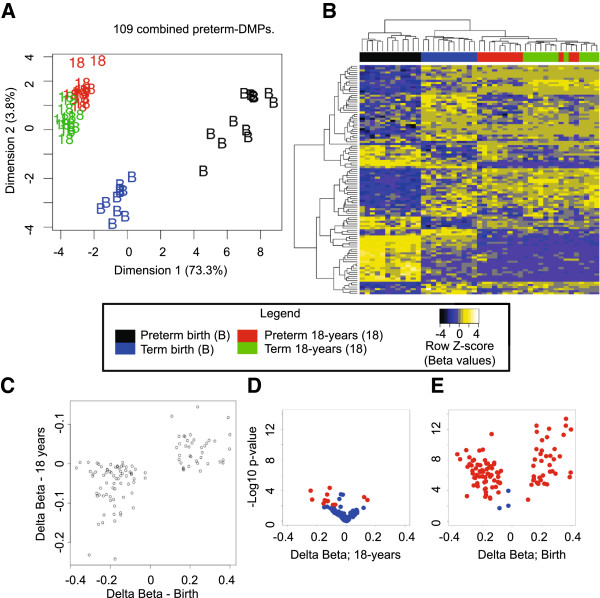
**Differentially methylated probes (DMPs) associated with preterm birth identified using birth and 18-year blood spots. (A)** Multi-dimensional scaling (MDS) and **(B)** heatmap plots show 109 combined preterm DMPs (comparing preterm with term, combining birth and 18-year samples for both groups). **(C)** Scatterplot displaying mean methylation differences (δβ) of birth (x-axis) and 18-year (y-axis) samples. Volcano plots showing δβ (x-axis) versus nominal *P*-values (−log_10_ scale) of combined preterm DMPs, with red points denoting probes with absolute methylation difference of greater than 0.05 and nominal *P* < 0.01 at **(D)**18 years and **(E)** birth. The sample groups are color coded as follows: term birth, blue; preterm birth, black; term birth at 18 years, green; preterm at 18 years, red.

Testing for differential methylation between term and preterm individuals in 18 year samples failed to detect any significant probes after correction for multiple testing. To further examine the possibility of a persistent effect of preterm birth at both time points, we compared all preterm and term samples in the two groups (birth and 18-year samples combined for each group) and found 109 combined preterm DMPs at a genome-wide level of significance (adjusted *P* < 0.05) (see Additional file 9: Table S8). Using MDS and hierarchical clustering, we found that this probe set separated sample groups almost perfectly by age and preterm/term status (Figure 4A,B). Interestingly, eight of these probes were not called as significant using only the birth samples. Six of these eight probes showed a mean β difference of greater than 0.1 between the preterm and term groups at both time points, and are located at the *PCSK9*, *TRIM71*, *SLC44A4*, *GPC6*, and *NFYA* gene bodies and one intergenic site. Of the 109 combined preterm DMPs, 11 showed a mean difference of β > 0.05 at both time points (Figure 4C,D), including two intergenic probes targeting CpG sites 270 bp apart within a CGI shore and a site within the *TINAGL1* 3′ UTR. Intriguingly, the two intergenic persistent sites of methylation difference flank a binding site for the early growth response 1 (EGR1) transcription factor identified previously in erythroid cells (see Additional file 2: Figure S3) [56].

Sequenom EpiTYPER confirmed differential methylation of regions flanking the EGR1-binding site (*P* < 0.05) (see Additional file 2: Figure S4) and the *TINAGL* DMP (*P* < 0.05) (see Additional file 2: Figure S5). However, Sequenom did not detect significant differential methylation at a putative persistent DMP located at the *MAP3K8* gene (see Additional file 4: Table S3)*.* We speculate that this may be due to the probe region containing two annotated deletion/insertion genetic variants of unknown allele frequency (rs67613960 and rs71525594) that may have confounded methylation measures [53,57]. Thus, among the 109 significant combined preterm DMPs, we found a total of 10 putative persistent preterm DMPs, defined as sites showing mean methylation difference of β > 0.05 at both time points. Taken together, these results raise the possibility that a minority of genomic regions carry a long-term epigenetic legacy of preterm birth.

## Discussion

In this exploratory study, we examined DNA methylation profiles associated with very preterm birth (<31 weeks of gestation) using longitudinally collected blood sampled from newborns and 18-year-olds, both stored as dried blood spots. We identified methylation differences in birth samples at several gene loci previously reported to co-vary with gestational age. These results demonstrate the utility of archived birth blood spot DNA for methylation profiling, in accordance with other recent studies [48,49,52].

We found widespread differences in DNA methylation at birth in preterm infants compared to with term controls. In agreement with previous studies [23,28,58], our data revealed methylation changes in blood associated with age. Some of these methylation differences are likely to reflect cell composition or functional differences in blood between preterm and term neonates, and between birth and at 18 years of age. For example, preterm-associated methylation differences at birth coincide with hematological changes that are correlated with gestational age, such as leukocyte [59,60] and nucleated reticulocyte [61] content. Further, gestational and age-related methylation changes may also reflect maturation of blood cells, including innate cytokine and adaptive immune induction [62-64]. Methylation change driven by these effects is evidenced by over-representation of birth DMPs and age DMPs in gene networks involved in hematological system development (see Additional file 4: Table S3; see Additional file 6: Table S5; see Additional file 7: Table S6).

Blood cell composition is well established as a predictive factor for inter-individual methylation variation in whole blood [65,66]. However, this variable does not readily explain the significant overlap in differentially methylated CpGs between preterm and term subjects at birth and also between birth and 18 years of age. We found 159 sites that showed increased methylation and 141 that showed decreased methylation from preterm to term birth and subsequently up to adulthood, suggesting a continuum of methylation change from mid-gestation to young adulthood for some regions of the genome (Figure 2). Genes associated with these sites were over-represented by direct targets of the upstream transcription factors NLRC5 and CIITA, master regulators of the MHC I-dependent [67-69] and MHC II-dependent [70,71] immune responses, respectively. These observations are consistent with gestational and post-natal changes to the immune system, during which time MHC responses are initiated [72]. We also found that genes associated with a continuum of methylation change were over-represented in embryonic development/morphogenesis and nervous system development, consistent with previous reports [73,74]. Teschendorff and co-workers have previously reported a correlation between age-associated loss of methylation and blood composition, but not with age-associated increases in methylation [58]. However, our data cannot disentangle cell-type effects. Other contributing factors may include developmental processes such as age-related changes to the progenitor cell pool [75-77], as suggested previously [28], or age-related shifts in blood cell signaling and metabolism [78].

We identified 431 CpG sites that changed in the opposite direction from preterm to term birth compared with birth to 18 years of age (Figure 2). Previous studies in human prefrontal cortex illustrate non-linear and directional changes in DNA methylation [27] and gene expression [79] during aging, suggesting that such changes are part of normal development. Our data defining CpG methylation sites that change direction during gestation and subsequently during post-natal life may reflect a distinctive methylation signature in the blood of term neonates. We speculate that these observations may reflect cell composition or functional differences in blood cells that are evidently unique to neonates born at full term [62,80,81].

We found very similar genomic contexts enriched in birth DMPs and age DMPs. Both showed over-representation of rDMRs and under-representation of CGIs and promoter regions. These results are consistent with findings from cross-sectional studies in adult mice [82] and humans [25,74], and with longitudinal studies of early post-natal life [23,83,84]. Taken together, these data indicate that similar regions of the genome are preferentially subject to epigenetic change during the second half of gestation, and during post-natal life in blood, and that these sites overlap rDMRs associated with *in vitro* pluripotency reprogramming. Although our genomic context enrichment data on differential methylation at 18 years used nominally significant methylation differences, our results suggest that inter-individual differences in methylation are more likely to occur in regions of the genome not associated with aging. These findings also suggest that gestation-related and age-related changes are unlikely to relate to ‘epigenetic noise’ [85]. However, we cannot determine if these observations reflect inter-individual blood composition differences, inter-individual DNA methylation variation, or associations with preterm birth.

At 18 years of age, most methylation differences identified in preterm babies are resolved, as evidenced by the lack of genome-wide significance in differential methylation at this time point. This is consistent with our conclusion that developmental changes and cell composition are the main components of methylation variation associated with birth DMPs and age DMPs. However, comparing preterm and term group analysis of birth and 18-year samples identified 109 statistically significant DMPs. Interestingly, eight of these CpG sites were not significantly differentially methylated at birth, suggesting that a larger sample size may indeed detect a long-term epigenetic legacy of preterm birth at a single time point. We observed persistently altered CpG methylation at *PCSK9*, *TRIM71*, *SLC44A4, GPC6*, and *NFYA* gene loci and at two intergenic CpG sites flanking a binding site for the EGR1 transcription factor. Taken together, these observations raise the possibility that persistent DNA methylation differences reflect a long-term legacy of preterm birth.

Limitations of the study include confounding factors related to inter-individual variation in blood composition, which may restrict power to detect birth DMPs and age DMPs. Our exploratory study requires replication in a larger cohort. This is particularly important to confirm the persistent epigenetic legacy of preterm birth identified in this report. Use of term-equivalent samples from preterm subjects would be useful in this context. Furthermore, statistical methods for deconvoluting mixed cell types [66] or adjustment for age [86] have not been described in context of gestation or neonatal development. Therefore, further studies addressing methylation differences in sorted cells during gestation, at full-term birth, and later in life may provide empirical data necessary to account for these confounders, as suggested previously by Houseman and colleagues [87].

## Conclusions

We report the first analysis of genome-scale methylation profiling using longitudinally collected archived blood spot DNA comparing very preterm and term subjects. We identified preterm birth-associated methylation differences at birth and demonstrated that these are mostly resolved by 18 years of age. We also described methylation changes that show a continual change from mid-gestation to young adulthood, and those that possibly reverse their direction of change. Finally, we found a minority of genomic sites that show persistent methylation differences between terms and preterms at both time points. These results suggest that a significant, long-term legacy of preterm birth might be observed using a larger sample size. Further work is required to examine if preterm birth-associated methylation differences co-vary with long-term health outcomes, early medical interventions, and/or genetic polymorphisms.

## Abbreviations

CGI: CpG island; DMP: Differentially methylated probe; DMR: Differentially methylated region; ES: Embryonic stem; FDR: False discovery rate; GA: Gestational age; GC: Guthrie card; GEO: Gene Expression Omnibus; IDAT: Intensity data; IPA: Ingenuity Pathways Analysis; MDS: Multi-dimensional scaling; O/E: Observed/expected; rDMR: Reprogramming-specific differentially methylated region; SNP: Single nucleotide polymorphism; SWAN: Subset-quantile within-array normalization; UTR: Untranslated region; VMP: Variable methylation probe.

## Competing interests

The authors declare that they have no competing interests.

## Authors’ contributions

MNC, AO, CT, PGD, PS, RS, LWD, and JMC were responsible for study design. MNC performed sample preparation and data analysis, and drafted the manuscript. AO oversaw analysis and interpretation of data, and DM performed additional analysis. CT, PGD, PS and LWD provided interpretation in the context of obstetrics and neonatology. LWD provided funding. YD performed locus-specific methylation analysis. JMC assisted in drafting the manuscript. All authors critically revised the manuscript and have read and approved the manuscript for publication.

## Supplementary Material

Additional file 1: Table S1 Oligonucleotide sequences of bisulfite-specific PCR and Sequenom MassArray primers. Click here for file

Additional file 2: Figure S1 Multi-dimensional scaling plot of sample relations based on all 347,789 probes. The relationship between DNA methylation of samples is shown with the four groups of samples color coded as follows: term birth, blue; preterm birth, black; term birth at 18 years, green; preterm at 18 years, red. **Figure S2.** DNA methylation β density plot of birth and 18-year longitudinal samples. Bimodal distribution of DNA methylation β-values in birth and 18-year samples. **Figure S3.** Sequenom and Infinium HM450 comparison of birth **differentially methylated probes (DMPs)** targeting the *VWF* gene body enhancer. **(A)** Methylation data from HM450 probe targets (red) and nearest analysable Sequenom EpiTYPER CpG unit (blue) from a single amplicon encompassing both HM450 probe targets. **(B)** Partial Sequenom amplicon sequence annotation displayed with CpGs/CpG units highlighted in the same colors. **Figure S4.** Genomic landmark and Sequenom analysis of long-term DMPs flanking tandem EGR1 consensus sites. **(A)** Methylation data from HM450 probe targets (red) and nearest analysable Sequenom EpiTYPER CpG unit (blue) from two separate amplicons each encompassing one HM450 probe target. **(B)** Partial Sequenom amplicon sequence annotation displayed with CpGs/CpG units from each amplicon highlighted in the same colors except for amplicon 7b CpG14 which is coincident with HM450 cg18598117. **(C)** Location of Infinium HM450 probes in relation to genomic landmarks including EGR1 chromatin immunoprecipitation sequencing (ChIP-seq) data, RNA sequencing (RNA-seq) reads and DNA methylation from human frontal cortex specimens derived from the UCSC browser. **Figure S5.** Sequenom analysis of long-term DMP at *TINAGL1* 3′UTR. **(A)** Methylation data from HM450 probe targets (red) and nearest analysable Sequenom EpiTYPER CpG unit (blue) from a Sequenom amplicon encompassing the target of probe cg06730678 (red). **(B)** Partial Sequenom amplicon sequence annotation displayed with CpGs/CpG units highlighted in the same colors. Click here for file

Additional file 3: Table S2 Birth differentially methylated probes. Click here for file

Additional file 4: Table S3 Gene ontologies from DAVID and Ingenuity Pathways Analysis (IPA) analysis using gene lists associated with birth differentially methylated probes (DMPs). Click here for file

Additional file 5: Table S4 Birth to 18 years (age) differentially methylated probes with β > 0.2. Click here for file

Additional file 6: Table S5 Gene ontologies from DAVID and Ingenuity Pathways Analysis (IPA) analysis using gene lists associated with age differentially methylated probes (DMPs). Click here for file

Additional file 7: Table S6 Gene ontologies from DAVID and Ingenuity Pathways Analysis (IPA) analysis using gene lists associated with directionally correlated birth and age differentially methylated probes (DMPs). Click here for file

Additional file 8: Table S7 Gene ontologies from DAVID and Ingenuity Pathways Analysis (IPA) analysis using gene lists associated with directionally opposed birth and age differentially methylated probes (DMPs). Click here for file

Additional file 9: Table S8 Combined preterm birth differentially methylated probes (DMPs): 109 significant combined preterm birth DMPs with adjusted *P* < 0.05 using birth and 18 year samples. False discovery rate (FDR)-adjusted and nominal birth and 18-year *P*-values are shown. Click here for file
