# Supplementary material for: Analysis of epigenetic changes in survivors of preterm birth reveals the effect of gestational age and evidence for a long term legacy
Source: Genome Med. 2013 Oct 18;5(10):96. doi: 10.1186/gm500 (PMC3978871; doi:10.1186/gm500)
Supplement: Additional file 2: Figure S1 — Multi-dimensional scaling plot of sample relations based on all 347,789 probes. The relationship between DNA methylation of samples is shown with the four groups of samples color coded as follows: term birth, blue; preterm birth, black; term birth at 18 years, green; preterm at 18 years, red. Figure S2. DNA methylation β density plot of birth and 18-year longitudinal samples. Bimodal distribution of DNA methylation β-values in birth and 18-year samples. Figure S3. Sequenom and Infinium HM450 comparison of birth differentially methylated probes (DMPs) targeting the VWF gene body enhancer. (A) Methylation data from HM450 probe targets (red) and nearest analysable Sequenom EpiTYPER CpG unit (blue) from a single amplicon encompassing both HM450 probe targets. (B) Partial Sequenom amplicon sequence annotation displayed with CpGs/CpG units highlighted in the same colors. Figure S4. Genomic landmark and Sequenom analysis of long-term DMPs flanking tandem EGR1 consensus sites. (A) Methylation data from HM450 probe targets (red) and nearest analysable Sequenom EpiTYPER CpG unit (blue) from two separate amplicons each encompassing one HM450 probe target. (B) Partial Sequenom amplicon sequence annotation displayed with CpGs/CpG units from each amplicon highlighted in the same colors except for amplicon 7b CpG14 which is coincident with HM450 cg18598117. (C) Location of Infinium HM450 probes in relation to genomic landmarks including EGR1 chromatin immunoprecipitation sequencing (ChIP-seq) data, RNA sequencing (RNA-seq) reads and DNA methylation from human frontal cortex specimens derived from the UCSC browser. Figure S5. Sequenom analysis of long-term DMP at TINAGL1 3′UTR. (A) Methylation data from HM450 probe targets (red) and nearest analysable Sequenom EpiTYPER CpG unit (blue) from a Sequenom amplicon encompassing the target of probe cg06730678 (red). (B) Partial Sequenom amplicon sequence annotation displayed with CpGs/CpG units highlighted in the same colors. [file gm500-S2.pdf]

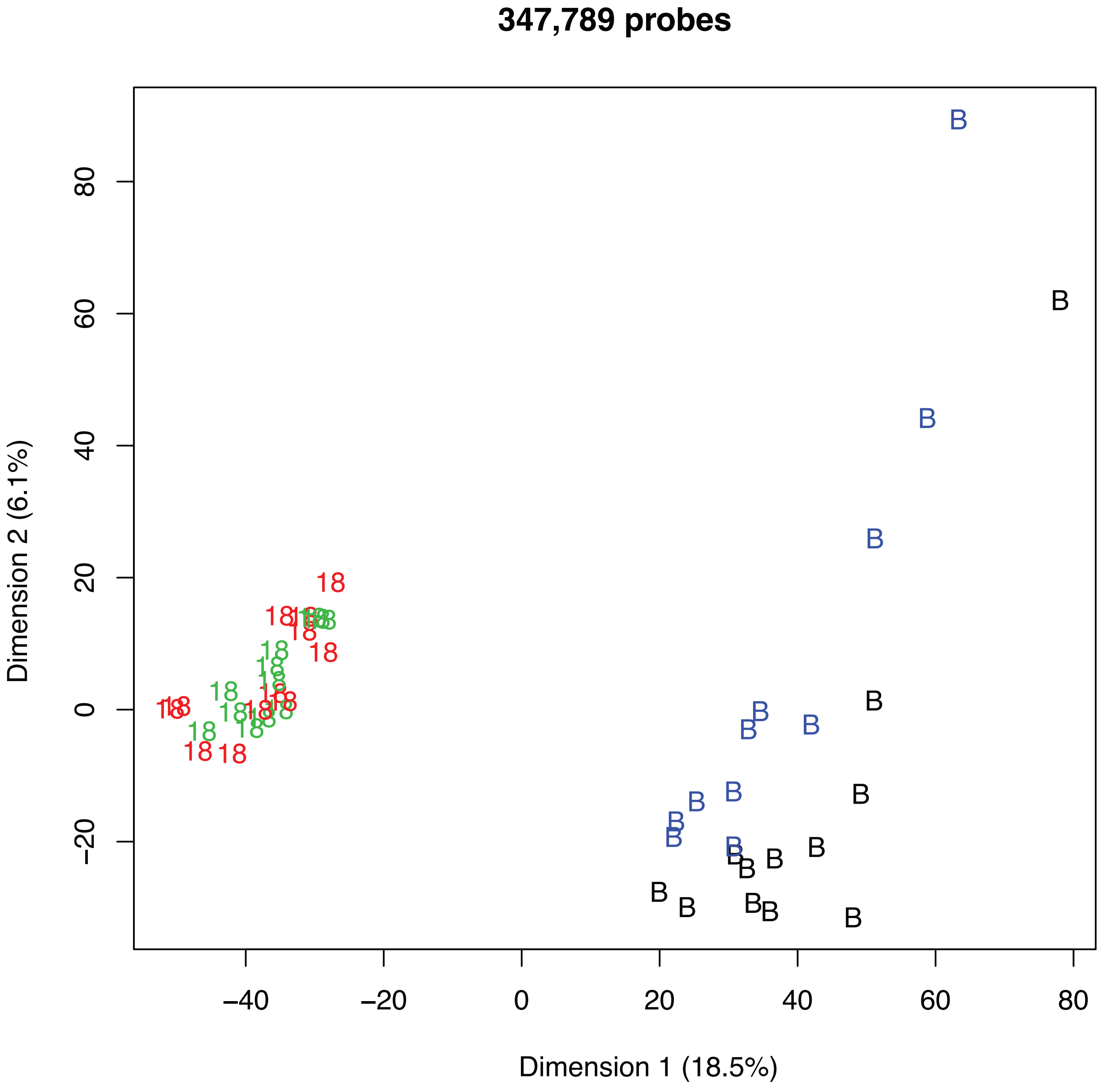

Figure S1. Multidimensional scaling plot of DNA methylation data from all samples and all 347,789 probes

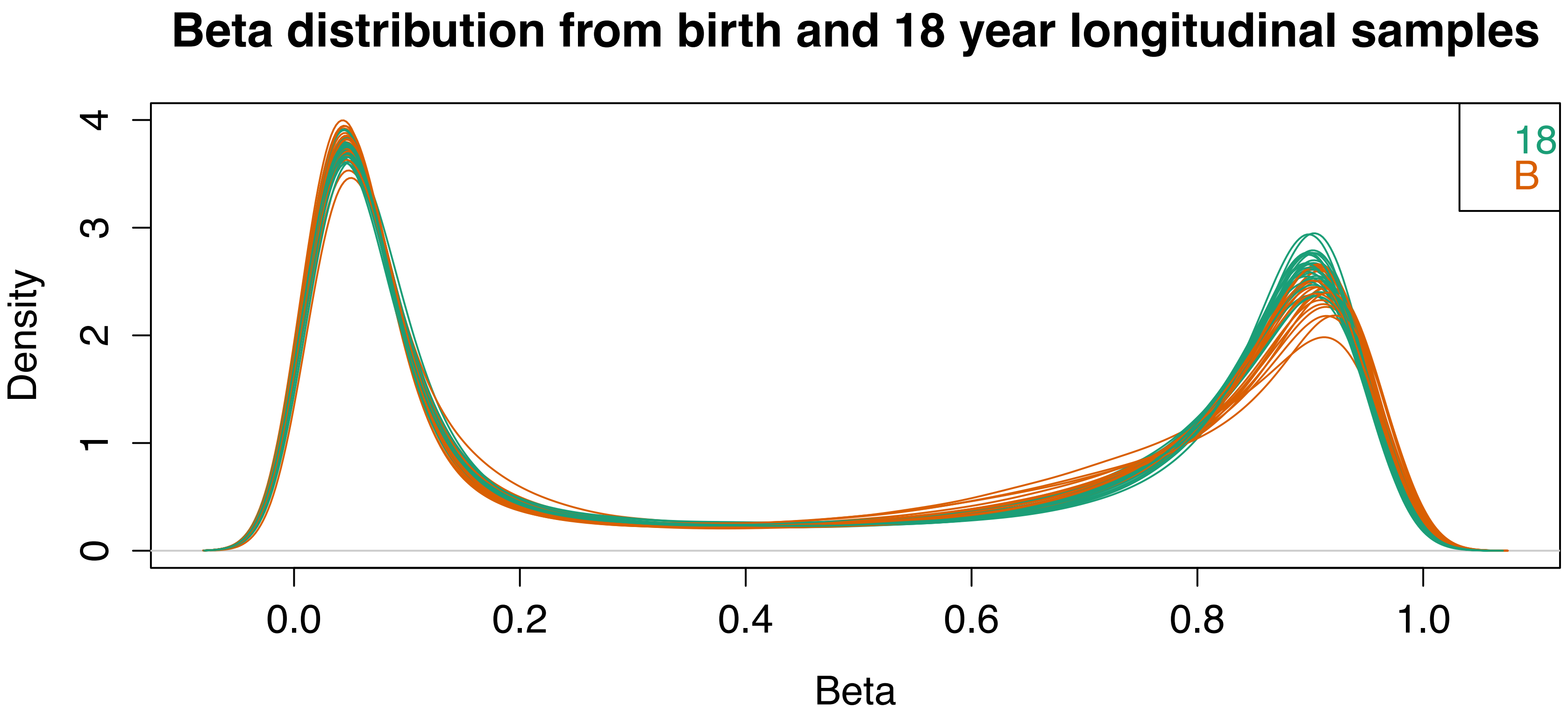

Figure S2. Beta distribution plots coloured by sample age

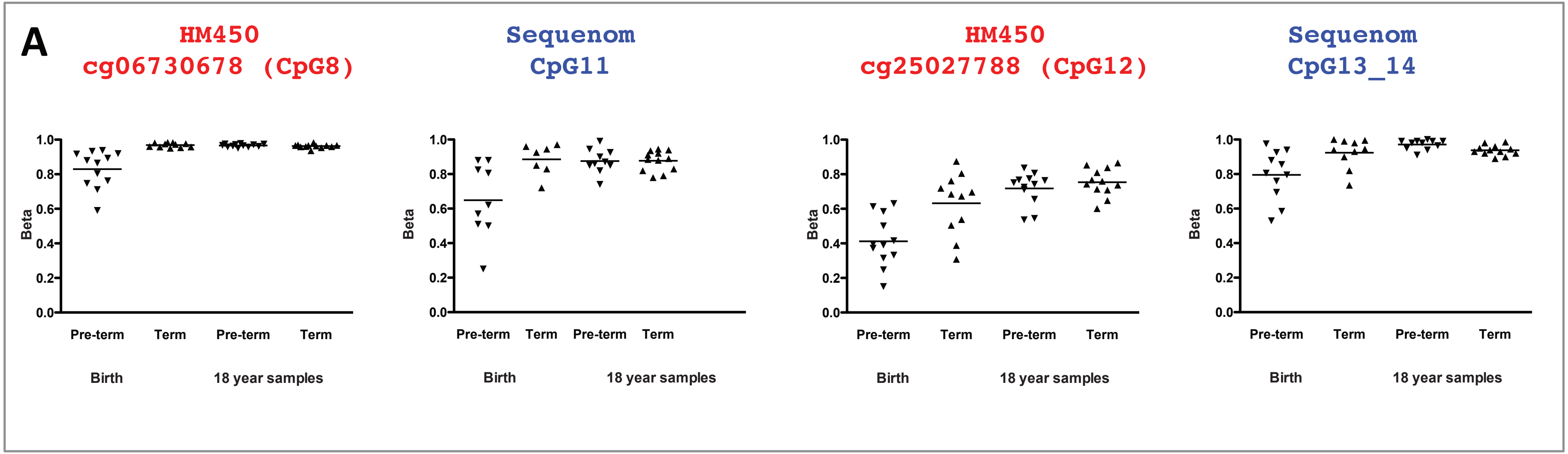

B

TGCCTCCGGAACGCACTGCACTAATGTGGAGACCTCGAGATTCTGCGAGGTCCCTGCCTTGCCCCCGGGTTCACATACTCATGCGCGGGTTGAG

Figure S3. Sequenom validation of VWF gene body enhancer birth-DMP

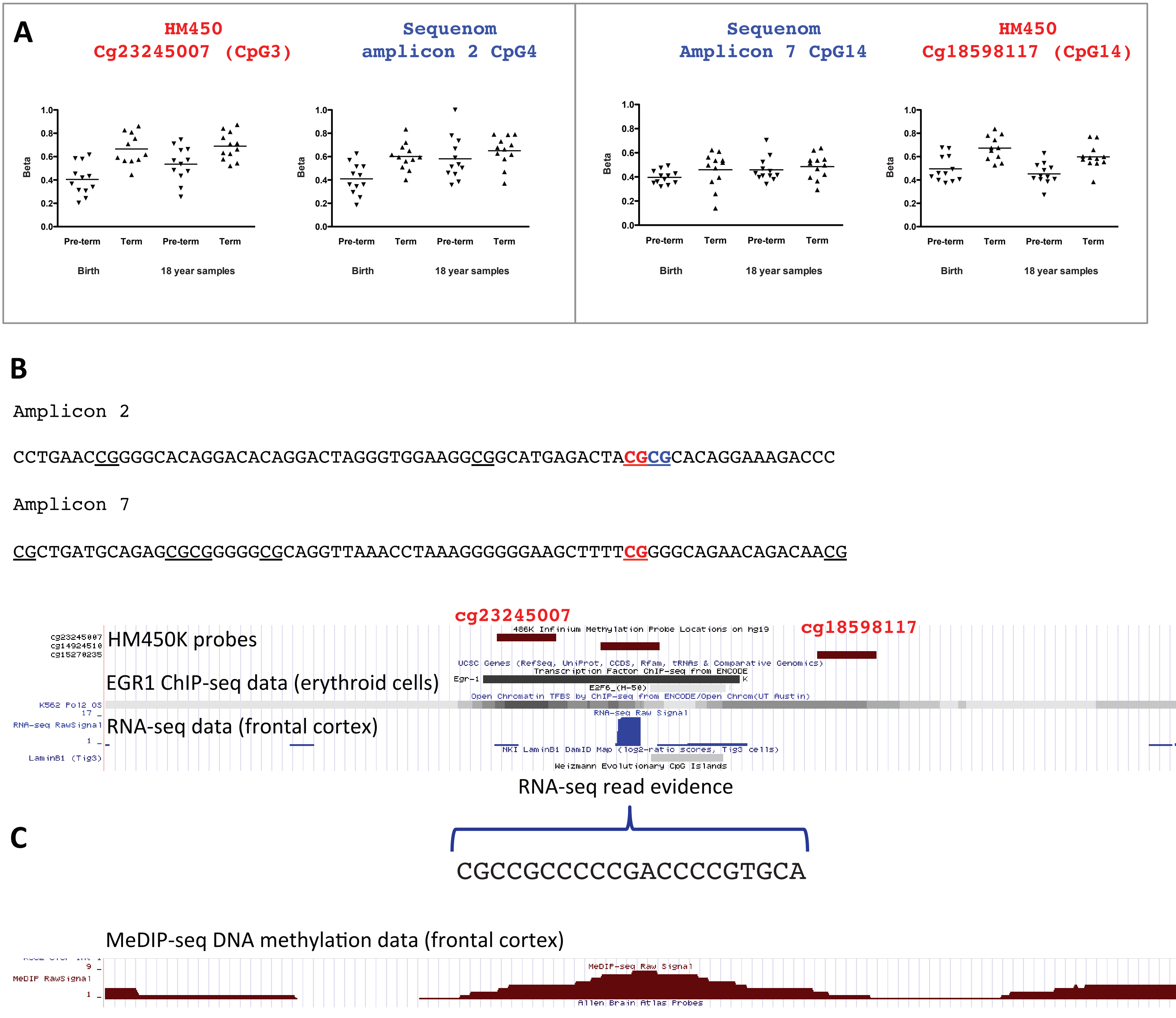

Figure S4. Sequenom validation of candidate long-term preterm-DMPs flanking tandem EGR1 consensus sites and genomic landmark context

A

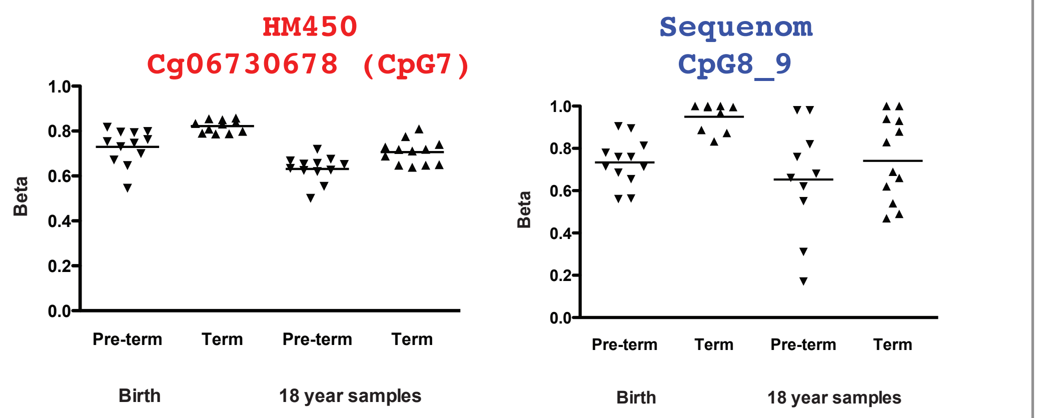

B

CCACGCGGGGTCCGGCCTGGGATCCAGGCTAAGGGCCGGCGGAAGAGGCCCCAATGGGGCGGTGACCCCAGCCTCGCCCGA

Figure S5. Sequenom validation of *TINAGL* 3’UTR candidate long term preterm-DMP
